# Meta-Analysis of Risk Stratification of SCN5A With Brugada Syndrome: Is SCN5A Always a Marker of Low Risk?

**DOI:** 10.3389/fphys.2019.00103

**Published:** 2019-02-19

**Authors:** Yihan Yang, Dan Hu, Frederic Sacher, Kengo F. Kusano, Xinye Li, Hector Barajas-Martinez, Mélèze Hocini, Yanda Li, Yonghong Gao, Hongcai Shang, Yanwei Xing

**Affiliations:** ^1^Guang'anmen Hospital, Chinese Academy of Chinese Medical Sciences, Beijing, China; ^2^Beijing University of Chinese Medicine, Beijing, China; ^3^Department of Cardiology and Cardiovascular Research Institution, Renmin Hospital of Wuhan University, Wuhan, China; ^4^Hôpital Cardiologique Haut Lévêque, Lyric institute, Université de Bordeaux, Bordeaux-Pessac, France; ^5^Division of Arrhythmia and Electrophysiology, Department of Cardiovascular Medicine, National Cerebral and Cardiovascular Center, Osaka, Japan; ^6^Global Genetics Corp, Ventura, CA, United States; ^7^Key Laboratory of Chinese Internal Medicine of the Ministry of Education, Dongzhimen Hospital Affiliated to Beijing University of Chinese Medicine, Beijing, China

**Keywords:** *SCN5A*, brugada syndrome, electrophysiology study, arrhythmia, sudden cardiac death

## Abstract

**Background:** *SCN5A* with Brugada syndrome (BrS) is not commonly considered as an independent risk marker for subsequent cardiac events. However, the risk of *SCN5A* combined with other clinical characteristics has not been fully investigated.

**Objectives:** The aim of this study is to investigate and evaluate risk stratification and related risk factors of *SCN5A* in BrS.

**Methods:** The databases of PubMed, EMBASE, Cochrane Library, MEDLINE, Chinese National Knowledge Infrastructure (CNKI) and Wanfang Data were searched for related studies published from January 2002 to May 2018 followed by meta-analysis. The BrS patients who underwent *SCN5A* gene tests were included. The prognosis and risk stratification of *SCN5A* combined with symptoms and asymptoms diagnosis in BrS, electrophysiology study (EPS) were then investigated and evaluated. Outcomes were defined as ventricular tachycardia/fibrillation (VT/VF), sudden cardiac death (SCD).

**Results:** Eleven suitable studies involving 1892 BrS patients who underwent *SCN5A* gene tests were identified. *SCN5A* (+) was not considered to be a significant predictor of future cardiac events (95% CI: 0.89–2.11; *P* = 0.15; *I*^2^ = 0%). However, *SCN5A* (+) patients with symptoms at diagnosis revealed a higher prevalence of future VT/VF, SCD compared to *SCN5A* (–) patients with symptoms at diagnosis. (95% CI: 1.06–3.70; *P* = 0.03 *I*^2^ = 0%) Among asymptomatic patients, the risk did not significantly differ between *SCN5A* (+) patients and *SCN5A* (–) patients. (95% CI: 0.51–4.72; *P* = 0.45 *I*^2^ = 0 %). In an investigation involving patients in EPS (–) BrS electrocardiogram (ECG), the risk of *SCN5A* (+) is higher than that of *SCN5A* (–) (*P* < 0.001).

**Conclusions:** In BrS patients with symptoms at diagnosis or EPS (–), the meta-analysis suggests that *SCN5A* (+) are at a higher risk of arrhythmic events than *SCN5A* (–).

## Introduction

BrS is an inheritable arrhythmogenic syndrome in a structurally integrated heart. According to current guidelines, it is the features of an ST segment elevation in the precordial leads which is related to improved danger of SCD (Priori et al., 2013). *SCN5A* gene mutation, as a risk factor for BrS, its prognostic significance in the general population remains controversial. On one hand, the present guideline shows that *SCN5A* mutation status cannot be an independent predictor of future cardiac events (Priori et al., 2013). On the other hand, BrS patients with *SCN5A*-mediated have higher prevalence of incidences of bradyarrhythmia events and conduction abnormalities (Yamagata et al., 2017). Recently, an important study particularly reported that *SCN5A* was the only gene which is clinically associated with BrS among the 21 included genes (Hosseini et al., 2018). Therefore, we initially preformed a comprehensive systematic review and meta-analysis of published data to elucidate the effect on mutations in *SCN5A* with symptoms and EPS, among the patients with BrS.

## Methods

### Search Strategy and Inclusion Criteria

A comprehensive literature research on MEDLINE, Embase, CNKI, and Wanfang Data databases was performed by two investigators. We used the query terms “Brugada syndrome” and “*SCN5A* Mutation” to identify and retrieve all potentially relevant studies from January 2002 to May 2018. Only full-size English articles published in peer-reviewed journals were considered for this meta-analysis. Studies were considered to be suitable whether they met the following criteria:

- the study was a prospective or retrospective observational study;
- inclusion of subjects with BrS were as previously defined;
- inclusion of patients who underwent *SCN5A* gene tests;
- the follow- up duration was long enough that the arrhythmia events would be observed;
- endpoint events [appropriate implantable cardioverter-defibrillator therapy (ICD), VF/VT, and SCD] were clearly defined;
- patients with endpoint events were clearly identified if they had *SCN5A* mutations;
- risk ratio (RR), hazard ratio (HR), odds ratio (OR), corresponding 95% confidence intervals (CIs), or necessary original data were presented.

In addition, we also contacted several corresponding authors of the studies to obtain more specific experimental data which were not included in the articles. Studies which demonstrated on only compound endpoints but particular data on all-cause mortality or different patient groups were not taken into account. In order to resolve the disagreements or uncertainties between the two investigators, a third investigator was responsible for rechecking the source data and consultation.

### Data Extraction

The elements of the extracted data were included in the meta-analysis: (a) publication information: surname of first author, publication year, and location; (b) type of study: multi-center or single-center study; (c) study design; (d) follow-up duration; (f) endpoint events (arrhythmic events were defined as VT/VF, SCD, and the combination of those two during the follow-up); (g) the quality score; (h) the characteristics of the population comprising sample size, gender, age, number of subjects with and without cardiac events, number of subjects with history of sudden cardiac arrest (SCA), syncope and family history of SCD. It also included the number of subjects with ICD, number of subjects with spontaneous type 1 ECG, and non-spontaneous type 1 ECG, number of symptomatic subjects with spontaneous type 1 ECG and non-spontaneous type 1 ECG, number of subjects who underwent EPS, the number of subject with EPS positive and EPS negative, number of EPS positive subjects who underwent ex-stimulation from 1 to 3 times, number of subjects with atrial fibrillation (AF) positive, number of subjects who underwent *SCN5A* gene test, number of subjects with *SCN5A* positive and *SCN5A* negative, number of symptomatic subjects with *SCN5A* positive and *SCN5A* negative during follow-up, number of subjects with Fragmented QRS (f-QRS) positive and f-QRS negative, number of subjects with early repolarization (ER) positive and ER negative; (i) among *SCN5A* (+) subjects with future cardiac events, the number of male subjects and female subjects, the number of subjects with or without family history of SCD, spontaneous type 1 ECG, symptoms and documented AF; (j) among *SCN5A* (–) subjects with future cardiac events, the number of male subjects and female subjects, the number of subjects with or without family history of SCD, spontaneous type 1 ECG, and symptoms and documented AF.

### Quality Assessment

The Methodological Index for Non-Randomized Studies (MINORS) was applied used for the Methodological quality to assess all studies. The use of maximum 24 points (each item scored from 0 to 2) was based on the following aspects: aim of the study, inclusion of consecutive patients, prospective data collection, appropriate endpoint to the aim of the study, unbiased evaluation of endpoints, follow-up period appropriate to the end-point, loss to follow-up no more than 5%, comparable control group, contemporary groups, base-line equivalence of groups, prospective calculation of the sample size, and use of adequate statistical analysis. After two independent investigators valued the included publications, the mean MINORS score was assessed as the final result. Studies were considered to be of low quality and high quality according to their MINORS scores of < 16 and ≥16 points, respectively (Slim et al., 2003).

### Statistical Analysis

A series of meta-analysis were performed including an analysis of all the patients who underwent *SCN5A* gene test and 8 subgroups, using Review Manager, version 5.3.5 (Revman; The Cochrane Collaboration, Oxford, U.K.). The concludes of the cardiac events outcome are indicated as ORs with 95% CIs for each study. To assess the heterogeneity among studies, the I^2^ value from the chi-square test was used, which describes the percentage of the variability in effect estimates due to heterogeneity, rather than sampling error. An I^2^ >50% indicates at least moderate statistical heterogeneity (Higgins et al., 2003).

We extracted data from 3 studies to compare categorical variables applying either a chi-square test or Fisher test (Sacher et al., 2013; Tokioka et al., 2014; Yamagata et al., 2017). TheSPSS 17.0 statistical package (SPSS Inc., IL, USA) was used to perform the analysis. In each analysis, statistical significance for treatment effect was defined at *P* < 0.05.

## Results

### Study Selection

**Figure 1:**
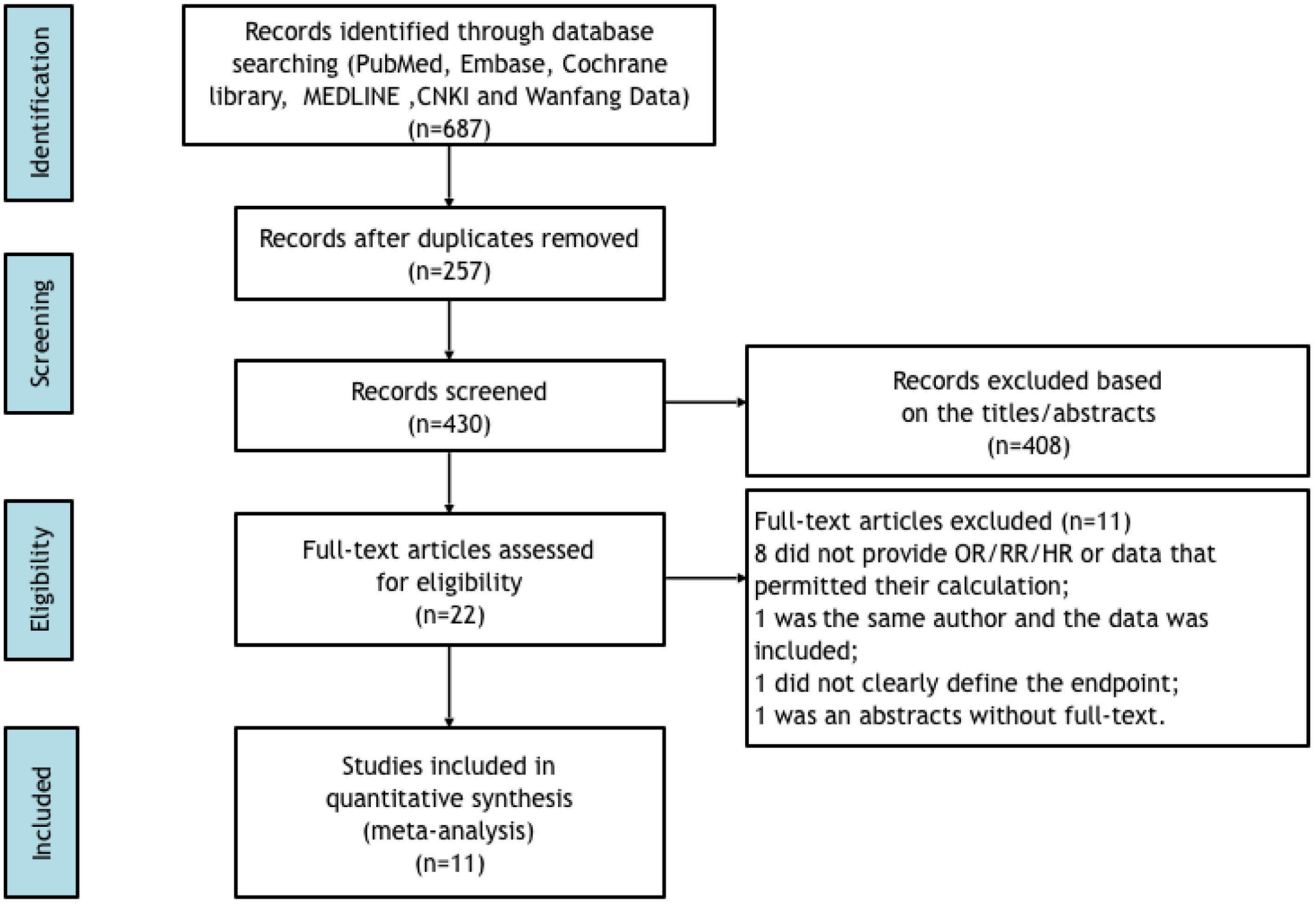
Flow diagram of data search and study selection.

A flow chart of the data research and study selection is shown in Figure 1. We excluded 257 duplicate studies across the number of 687 records that were identified by our research criteria. After screening the titles and abstracts, 408 studies were into the discard since they were categorized as guidelines, editorials, case reports, review articles, animal studies, laboratory studies, or unrelated to the present study. Then, 22 potential relevant studies were retrieved for specific evaluation. Of these, a number of 11 studies were further excluded from further analysis because of the following reasons: 8 studies did not provide RRs, ORs, or HRs or data could be calculated, or the 95% confidence intervals; one study did not clearly define the type of abnormal QRS complex; one did not clearly define the endpoints, and one was only an abstract without full-text.

Eleven studies (six prospective and five retrospective) were ultimately involved in this meta-analysis constituting 1892 patients with BrS in total (Table 1) (Gasparini et al., 2002; Juang et al., 2003; Mok et al., 2004; Liang et al., 2006; Yuan et al., 2008; Priori et al., 2012; Sacher et al., 2013; Tokioka et al., 2014; Andorin et al., 2016; Calò et al., 2016; Yamagata et al., 2017). The average age of the BrS patients was from 11 to 53 years old. A spontaneous type 1 ECG pattern of BS was reported in 68.8% of patients and a *SCN5A* gene test was performed on 1075 patients (56.8%). A positive genetic mutation was demonstrated in 248 patients (23.1%). Among these, 41 patients (16.5%) were demonstrated to have symptoms. Whereas, a total of 127 patients (15.4%) out of 827 patients (76.9%) with a negative *SCN5A* gene mutation were symptomatic. The mean follow-up duration ranged from 20 to 77 months. During follow-up, 229 patients (12.1%) suffered an arrhythmia event (syncope, non-sustained VT, aborted sudden cardiac death, and appropriate ICD shocks caused by VT/VF). All involved studies were assessed as high-quality publications (average MINORS score: 15 ± 2.9). In addition, we pursued further analysis to attempt to establish the relationship among *SCN5A*, other clinical features and subsequent cardiac events. The clinical characteristics of the 698 BrS patients from 3 studies are summarized in Table 2 and **Figure 3** (Sacher et al., 2013; Tokioka et al., 2014; Yamagata et al., 2017). It consists of 660 male patients and 37 female patients. In the *SCN5A*+ and *SCN5A*– patient groups, 23 and 83 individuals experienced subsequent cardiac events, respectively. A total of 161 patients had a family history of SCD. A spontaneous type 1 BrS ECG was demonstrated in 72% of patients and 577 patients underwent EPS in total, with 329 patients displaying positive results. With regard to documented AF, 2 of the 100 AF positive patients and 82 of the 586 AF negative patients had arrhythmia events during follow-up.

**Table 1:** Study characteristics of 11 studies included in meta-analysis.

**Investigator**	**Location**	**Type of study**	**Study of design**	**Study population**	**Mean follow-up**	**Endpoint**	**Quality score**
Gasparini et al., 2002	Italy	SC	PS	Patients with BrS underwent a PES protocol	20 ± 12 months	PES protocol completion/induction of sustained/reproducible nonsustained fast ventricular arrhythmia	15
Juang et al., 2003	Taiwan	MC	RS	Patients with the diagnosis of the BrS	29 ± 17 months	Seizure/syncope/sudden cardiac death	14
Mok et al., 2004	Hong Kong	MC	PS	Patients with type 1 Brugada ECGs	25.8 ± 10.9monthes	Syncope/syncopal ventricular arrhythmia/sudden death/appropriate ICD shock	20
Liang et al., 2006	China	SC	PS	Patients with Brugada ECGs or suspected Brs	NA	Syncope/VT	13
Yuan et al., 2008	China	SC	PS	patients with Brugada ECGs	NA	Syncope/VT	8
Priori et al., 2012	Italy	MC	PS	Patients with type 1 ECGs, without history of cardiac arrest	36 ± 8 months	The occurrence of VF or appropriate ICD interventions	16
Sacher et al., 2013	France	SC	RS	Patients with type 1 Brugada ECGs withimplantable cardioverter-defibrillator	77 ± 42 months	Aborted sudden cardiac arrest/syncope	16
Tokioka et al., 2014	Japan	SC	RS	Patients with a Brugada-type ECG	45.1 ± 44.3 months	VF/SCD	16
Andorin et al., 2016	Europe	MC	RS	Patients with Brugada ECG under 19 years of age	54 months	Sudden death/documented VT or VF/appropriate ICD shock	15
Calò et al., 2016	Italy	MC	PS	Patients with spontaneous type 1 BrS ECG phenotype	48 ± 38.6 months	VF/SCD	16
Yamagata et al., 2017	Japan	MC	RS	Patients with type 1 Brugada ECG pattern	72 months	Documented atrial fibrillation/appropriate ICD interventions	16

*BrS, Brugada syndrome; ECG, electrocardiogram; ICD, implantable cardioverter defibrillator; PES, Programmed Electrical Stimulation; MC, multicenter study; NA, not available; n, number; PS, prospective study; RS, retrospective study; SC, single center study; SCD, sudden cardiac death; VF, ventricular fibrillation; VT, ventricular tachycardia*.

**Table 2:** Patients' characteristics of 11included studies.

	**Gasparini et al., 2002**	**Juang et al., 2003**	**Mok et al., 2004**	**Liang et al., 2006**	**Yuan et al., 2008**	**Priori et al., 2012**	**Sacher et al., 2013**	**Tokioka et al., 2014**	**Andorin et al., 2016**	**Calò et al., 2016**	**Yamagata et al., 2017**
Total Patients, *n*	21	10	50	4	7	308	378	246	106	347	415
Male/female, *n*	18/3	10/0	47/3	4/0	7/0	247/61	310/68	236/10	58/48	272/75	403/12
Age (years)	34	46 ± 7	53	40.9	43.6 ± 8.7	47 ± 12	46 ± 13	47.6 ± 13.6	11.1 ± 5.7	45 ± 13.1	46 ± 14
Symptomatic, *n* (%)	0 (0)	3 (30)	30 (60)	4 (100)	4 (57)	14 (4)	46 (12)	24 (10)	10 (9)	32 (9)	62 (15)
History of SCA, *n* (%)	1 (5)	9 (90)	8 (16)	NA	3 (43)	NA	31 (8)	13 (5)	NA	0 (0)	88 (21)
History of sycope, *n* (%)	8 (38)	1 (10)	12 (24)	2 (20)	4 (57)	65 (21)	181 (48)	40 (16)	15 (14)	14 (4)	99 (24)
Asymptomatic, *n* (%)	12 (57)	0 (0)	30 (60)	0 (0)	3 (43)	243 (80)	166 (44)	NA	85 (80)	316 (91)	228 (55)
Family history of SCD, *n* (%)	8 (38)	1 (10)	7 (14)	NA	3 (43)	NA	111 (30)	69 (28)	46 (43)	71 (20)	64 (15)
Patients with ICD, *n* (%)	NA	8 (80)	8 (16)	NA	NA	137 (44)	308 (81)	63 (26)	22 (21)	98 (28)	241 (58)
Spontaneous type1 ECG, *n* (%)	19 (90)	NA	43 (86)	NA	5 (71)	171 (56)	226 (60)	156 (63)	36 (34)	347 (100)	299 (72)
Events, *n* (%)	0 (0)	NA	17 (39)	NA	3 (60)	13 (8)	35 (15)	22 (14)	8 (22)	32 (10)	48 (16)
Non-Spontaneous type1 ECG, *n* (%)	2 (9.5)	NA	7 (14)	NA	0 (0)	NA	152 (40)	90 (37)	70 (66)	0 (0)	116 (28)
Events,*n* (%)	0 (0)	NA	3 (43)	NA	0 (0)	NA	11 (7)	2 (2)	2 (3)	0 (0)	14 (12)
Patients ungergo EPS, *n* (%)	21 (100)	8 (80)	30 (60)	NA	7 (100)	NA	310 (82)	155 (63)	NA	186 (54)	339 (82)
EPS+, (n)	18 (86)	6 (75)	19 (63)	NA	NA	NA	228 (73)	71 (46)	NA	77 (41)	191 (56)
up to 1 ex	0 (0)	NA	2 (10)	NA	NA	NA	NA	NA	NA	NA	NA
up to 2 ex	12 (67)	NA	9 (47)	NA	NA	NA	NA	NA	NA	NA	NA
up to 3 ex	6 (33)	NA	8 (42)	NA	NA	NA	NA	NA	NA	NA	NA
EPS-, *(n)*	3 (14)	2 (25)	11 (37)	NA	1 (14.3)	NA	82 (26)	84 (54)	NA	109 (59)	148 (44)
AF (+), *n* (%)	NA	NA	NA	NA	NA	NA	32 (8)	44 (17)	NA	NA	64
Patients undergo DNA tested, *n* (%)	21 (100)	4 (40)	36 (72)	4 (100)	7 (100)	123 (40)	160 (42)	123 (50)	75 (71)	107 (31)	415 (100)
*SCN5A* (+), *n* (%)	8 (38)	1 (25)	5 (14)	1 (25)	1 (14)	24 (20)	41 (26)	17 (26)	58 (77)	32 (30)	60 (14)
events, *n* (%)	0 (0)	1 (100)	2 (40)	1 (100)	0 (0)	3 (13)	6 (15)	4 (24)	9 (16)	2 (6)	13 (22)
*SCN5A* (−), *n* (%)	13 (62)	3 (75)	31 (86)	3 (75)	6 (86)	99 (80)	119 (74)	106 (74)	17 (23)	75 (70)	355 (86)
events, *n* (%)	0 (0)	2 (67)	18 (58)	3 (100)	4 (67)	6 (14)	16 (13)	19 (18)	0 (0)	10 (13)	49 (14)
f-QRS (+), *n* (%)	NA	NA	NA	NA	NA	25 (8)	NA	78 (32)	NA	85 (25)	NA
f-QRS (–), *n* (%)	NA	NA	NA	NA	NA	283 (92)	NA	158	NA	262 (76)	NA
ER (+), *n* (%)	NA	NA	NA	NA	NA	NA	NA	25 (10)	NA	30 (9)	NA
ER (–), *n* (%)	NA	NA	NA	NA	NA	NA	NA	221	NA	317 (91)	NA

*Data were presented as mean ± SD, median, or percentage where possible; SCA, sudden cardiac arrest; NA, not available; SCD, sudden cardiac death; ICD, implantable cardioverter defibrillator;ECG, electrocardiogram; EPS, electrophysiological study; n, number; ex, extrastimuli; f-QRS, fragmented QRS; ER, early repolarization*.

As for the symptomatic and asymptomatic BrS patients, we added an extra study based on the results from Andorin et al. and only did the meta-analysis (Andorin et al., 2016). Therefore, a total of 317 patients with symptoms at diagnosis and 456 patients without symptoms at diagnosis were identified. The symptoms were defined as patients with a history of ACA, SCD, or syncope.

### SCN5A (+) and SCN5A (–) Groups

Overall, BrS patients with a positive *SCN5A* gene mutation were not proven to be a significant predictor of future cardiac events (OR 1.37, 95% CI: 0.89–2.11, *P* = 0.15; Heterogeneity: *P* = 0.52, *I*^2^ = 0%, Supplementary Figure 1).

### Symptomatic at Diagnosis and Asymptomatic at Diagnosis Groups

**Figure 2:**
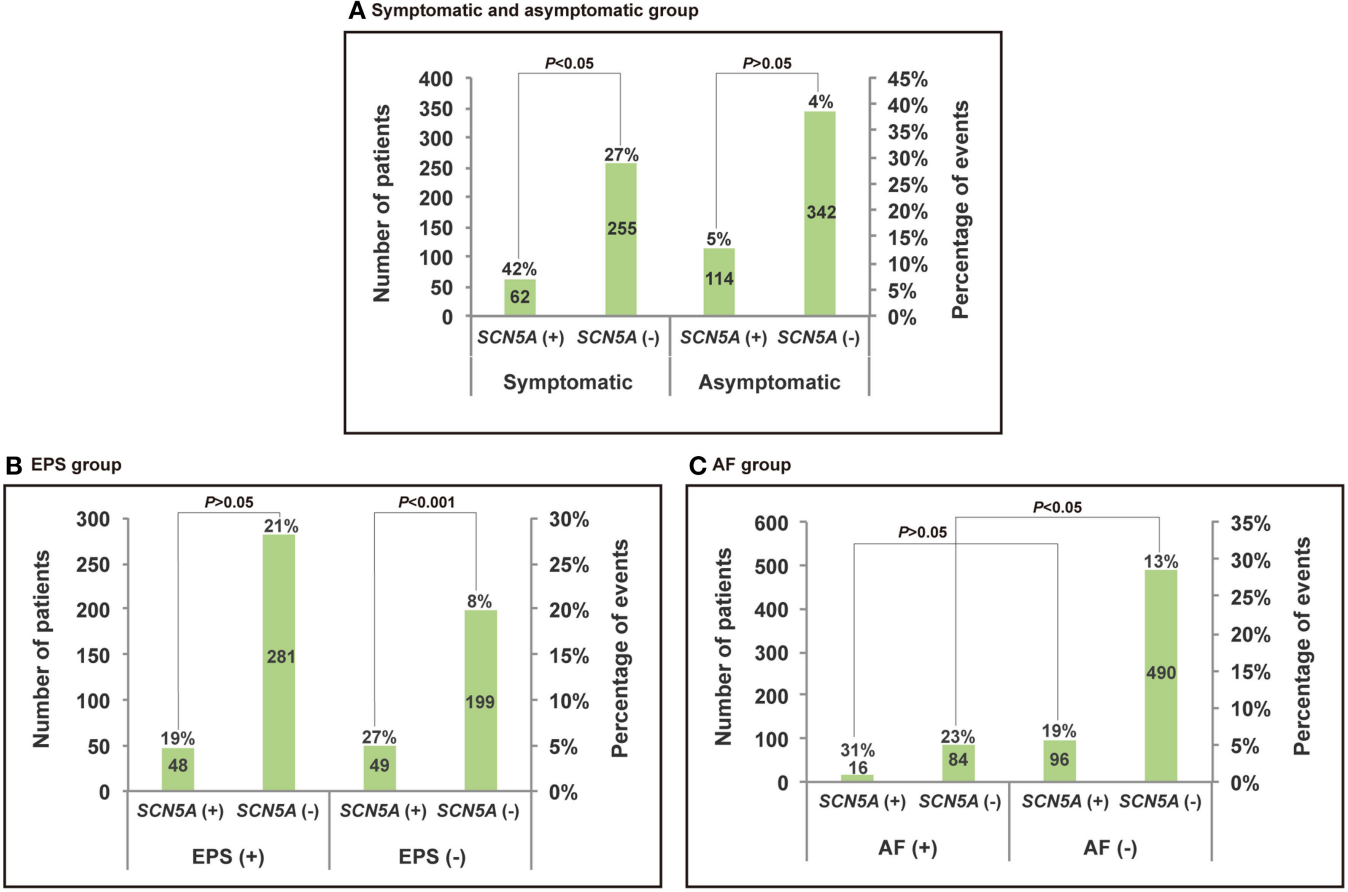
Histogram and broken line for the comparison between events of *SCN5A* (+) and *SCN5A* (–) of subgroups. **(A)** Symptomatic at diagnosis group. **(B)** EPS group. **(C)** AF group.

**Figure 3:**
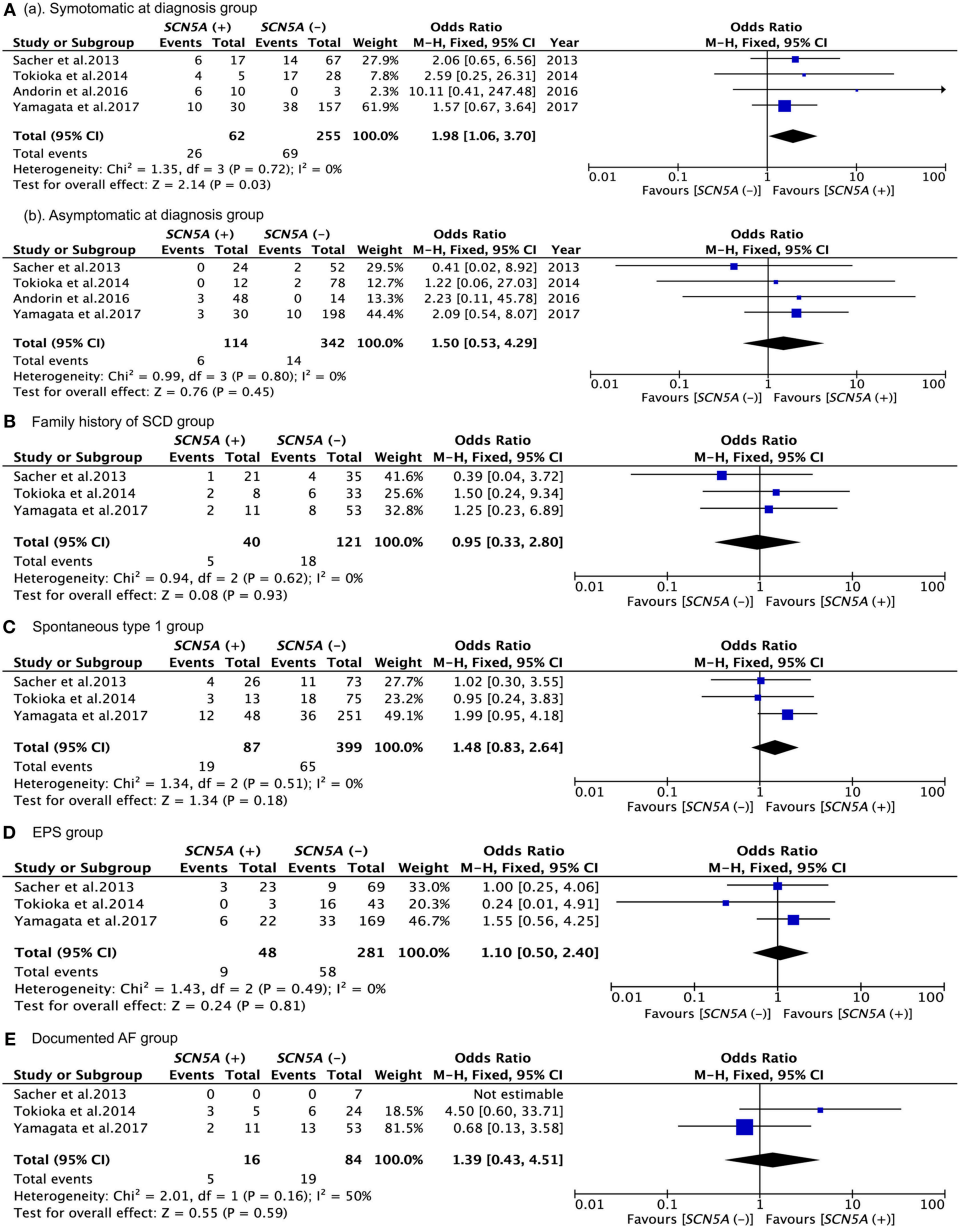
Forest plots comparing outcomes of subgroups. **(Aa)** Comparison between events of *SCN5A* (+) and *SCN5A* (–) in symptomatic at diagnosis group; **(Ab)** comparison between events of *SCN5A* (+) and events of *SCN5A* (–) in asymptomatic at diagnosis group; **(B)** comparison between events of *SCN5A* (+) and *SCN5A* (–) in family history of SCD group; **(C)** comparison between events of *SCN5A* (+) and *SCN5A* (–) in spontaneous type 1 group; **(D)** comparison between events of *SCN5A* (+) and *SCN5A* (–) in EPS group; **(E)** comparison between events of *SCN5A* (+) and *SCN5A* (–) in documented AF group.

The results of the analysis are presented in Figures 2, 3 and Table 3. According to the present meta-analysis, 26 (42%) of 62 (20%) *SCN5A* (+) patients and 69 (27%) of 255 (80%) *SCN5A* (–) patients had cardiac events. A total of 6 (5%) in 114 (25%) *SCN5A* (+) patients and 14 (4%) in 342 (75%) *SCN5A* (–) patients experienced future arrhythmic events (Figure 3) (Sacher et al., 2013; Tokioka et al., 2014; Andorin et al., 2016; Yamagata et al., 2017). In comparison with the asymptomatic at diagnosis patients (OR: 1,54, 95% CI: 0,51–4,72, *P* = 0.45; Heterogeneity: *P* = 0.62, *I*^2^ = 0 %, Figure 3), *SCN5A* (+) patients who were symptomatic at diagnosis displayed an increased risk of arrhythmic events. (OR 1,98, 95% CI: 1,06–3,70, *P* = 0.03; Heterogeneity: *P* = 0.72, *I*^2^ = 0%, Figure 3) (Sacher et al., 2013; Tokioka et al., 2014; Andorin et al., 2016; Yamagata et al., 2017).

**Table 3:** Comparison between subgroup of *SCN5A* (+) group and *SCN5A* (–) group.

			***SCN5A*** **(+)**	***SCN5A*** **(–)**	***P*****-value**
EPS status	Positive	Total	48	281	0.764
		Events	9	58	
	Negative	Total	49	199	^*^<0.001
		Events	13	15	
		*P*-value	0.360	^*^<0.001	
Spontaneous type 1 ECG	Positive	Total	87	399	0.215
		Events	19	65	
	Negative	Total	31	181	0.691
		Events	4	19	
		*P*-value	0.281	0.090	
Documented AF status	Positive	Total	16	84	0.495
		Events	5	19	
	Negative	Total	96	490	0.142
		Events	18	64	
		*P*-value	0.252	^*^0.021	

Bold value means statistically significant (

* *P < 0.05)*.

### Family History of SCD Group

The results of the analysis are presented in Figures 2, 3 and Table 3. During follow-up, 5 (13%) of 40 (25%) *SCN5A* (+) patients and 16 (13%) of 121 (75%) *SCN5A* (–) patients had arrhythmic events (Sacher et al., 2013; Tokioka et al., 2014; Yamagata et al., 2017). The meta-analysis result revealed that a family history of SCD had little influence on the incidence of future events among *SCN5A* (+) patients. (OR = 0.95, 95% CI: 0.33–2.80, *P* = 0.62; Heterogeneity: *P* = 0.93, *I*^2^ = 0%, Figure 3).

### Spontaneous Type 1 BrS ECG Groups

The results of the analysis are presented in Figure 3 and Table 3. Cardiac events were documented, respectively in 22% *SCN5A* (+) and 16% *SCN5A* (–) groups, with no significant difference for patients with spontaneous type 1 BrS ECG patterns (OR = 1.48, 95% CI: 0.83–2.64, *P* = 0.18; Heterogeneity: *P* = 0.51, *I*^2^ = 0%, Figure 3) (Sacher et al., 2013; Tokioka et al., 2014; Yamagata et al., 2017). A similar result was demonstrated for the chi-square test as well. (*P* = 0.215 vs. P = 0.691, Table 3).

### Electrophysiological Study Groups

The results of the analysis are presented in Figures 2, 3 and Table 3. *SCN5A* (+) and *SCN5A* (–) patients who were symptomatic during follow-up were presented in 19 and 21%, respectively (Figure 3) (Sacher et al., 2013; Tokioka et al., 2014; Yamagata et al., 2017). No statistically significance difference was revealed with respect to the patients with EPS positive between the *SCN5A* (+) group and the *SCN5A* (–) group. (OR = 1.12, 95 % CI: 0.51–2.44, *P* = 0.78; Heterogeneity: *P* = 0.50, *I*^2^ = 0%, Figure 3). According to the results from Table 3, *SCN5A* (–) BrS patients with positive EPS results had a higher prevalence for future arrhythmia events. (*P* = 0.764 vs. *P* < 0.001). Furthermore, *SCN5A* (+) patients with a negative EPS result had a higher prevalence of cardiac events compared to the case of for *SCN5A* (–) patients. (*P* = 0.0.360 vs. *P* < 0.001).

### Documented AF Groups

The results of the analysis are summarized in Figures 2, 3 and Table 3 (Sacher et al., 2013; Tokioka et al., 2014; Yamagata et al., 2017). During follow-up, 6 (31%) of 16 *SCN5A* (+) patients and 17 (23%) of 84 *SCN5A* (−) patients had arrhythmic (OR = 2,10, 95% CI: 0.69–6.39, *P* = 0.19; Heterogeneity: *P* = 0.13, *I*^2^ = 50 %, Figure 3) (Sacher et al., 2013; Tokioka et al., 2014; Yamagata et al., 2017). In comparison with *SCN5A* (–) patients with AF, no statistically significant difference was observed for *SCN5A* (+) patients with AF. (*P* = 0.495 vs. *P* = 0.142). Based on the results from Table 3, *SCN5A* (–) patients with documented AF had a higher rate of cardiac events compared to *SCN5A* (–) patients without AF (*P* = 0.021).

## Discussion

The meta-analysis yielded the following main findings: (a) the *SCN5A* gene mutation may not be associated with subsequent cardiac events; (b) *SCN5A* (+) patients with symptoms at diagnosis display a higher risk of arrhythmic events compared to *SCN5A* (–) patients with symptoms at diagnosis (c) Among EPS (–) BrS patients, *SCN5A* (+) patients have a higher prevalence of future cardiac events compared to *SCN5A* (–) patients. Compared with *SCN5A* (–) BrS patients with negative EPS results, *SCN5A* (–) BrS patients with positive EPS results had a higher prevalence for subsequent arrhythmia events.

The *SCN5A* gene locates on chromosome 3p21 and contains 28 exons spanning approximately 80 kb, and encodes α-subunit protein Na_V_1.5. In most situation, *SCN5A* mutations observed in BrS1 were loss-of-function mutations (Hedley et al., 2009), which results in the reduced availability of sodium channels, either by reducing trafficking and expression of channel on membrane surface, or through changing gate properties of channel (Remme, 2013). Variant mutations in *SCN5A* led to different mechanisms of action. Some mutations resulted in a reduced current density, I_Na_, while others did not result in a decrease in I_Na_. In a few cases, the picture was more complicated (Hedley et al., 2009). *SCN5A* gene mutation in particular regions also resulted in a worse outcome during follow-up. For example, pore regions documented by yamagata et al. were identified as being associated with a higher prevalence of future arrhythmia events (Yamagata et al., 2017).

### SCN5A (+) and SCN5A (–) Group

The analysis included a total number of 1,075 patients from 11 studies over 10 countries. In accordance with the majority of previous study results, a negative conclusion was obtained. According to the current guideline, a *SCN5A* (+) is not a risk marker for the occurrence of BrS. Genetic testing is not recommended without a diagnostic ECG; unless, there was a successfully genotyped proband observed in family members (Priori et al., 2013). Based on Calò et al. study, patients who developed VF or SCD displayed a lower rate of mutations in *SCN5A* gene (Andorin et al., 2016). The present subgroup analysis indicated a similar result. Also, the EPS results and T waves changes on ECG did not differ significantly between the *SCN5A* (+) patients and the *SCN5A* (–) patients (Tokioka et al., 2014; Tse et al., 2018). On the other hand, the conclusion presented by Andorin et al is that an absent *SCN5A* mutation probably led to a lower risk of subsequent cardiac events (Andorin et al., 2016). Furthermore, *yamagata et al*. demonstrated that *SCN5A* gene mutation positive was an independent risk marker for cardiac events among all probands by applying the Cox proportional hazards model (HR = 1.1, 95% CI: 1.1 to 3.8, *P* = 0.02) (Yamagata et al., 2017).

### Symptomatic at Diagnosis and Asymptomatic at Diagnosis Groups

Recently, Yamagata et al. demonstrated that *SCN5A* (+) probands, especially for mutation in the pore region presenting with prior ACA or syncope, were more likely to be associated with future cardiac events compared to *SCN5A* (–) BrS patients. However, no significant difference was revealed between asymptomatic *SCN5A* (+) probands and *SCN5A* (–) probands (Yamagata et al., 2017). In addition, a recent meta-analysis reported that symptomatic male BrS patients were at higher risk than asymptomatic male BrS patients (Yuan et al., 2018). Based on these results, we performed a further subgroup meta-analysis and extracted data from Yamagata et al. and three other studies involving 773 patients who underwent *SCN5A* gene test with either prior ACA or syncope. Interestingly, a significant difference was noted between the *SCN5A* (+) patients with symptoms at diagnosis and the *SCN5A* (–) patients with symptoms at diagnosis. With respect to BrS patients without symptoms at diagnosis, no significant difference was observed. *Sacher et al*. reported on a large multi-center registry on the outcome of BrS patients implanted with an ICD in France (Sacher et al., 2013). Tokioka et al. investigated the combination of ECG markers (depolarization and repolarization abnormalities) on risk assessment of VF in Japan. Both studies indicated that the role of the *SCN5A* mutation was of minor influence (Tokioka et al., 2014). Andorin et al. focused on BrS patients under age 19 at diagnosis in 16 European hospitals and concluded that there was a higher prevalence of *SCN5A* (+) pediatric patients with life-threatening arrhythmias (Andorin et al., 2016).

Some studies have reported that patients with *SCN5A* (+) had a higher prevalence of abnormalities in conduction (longer PQ interval, longer QRS duration, and frequent fragmentation). The decreased of sodium current reduces the action potential upstroke velocity, resulting in atrial and ventricular conduction deceleration accompanied by prolonged PR and QRS intervals (Remme, 2013). Moreover, there was sufficient background for the developed conduction abnormality leading to higher possibilities for arrhythmic events. Furthermore, it was confirmed that an abnormality in a cardiac ion channel may result in cell damage and death in patients with BrS. On this basis, it can be argued that the arrhythmic event may occur when a specific threshold of cell damage is reached, due to the severity of the ion channel protein mutation (Yamagata et al., 2017). The aforementioned facts may explain why *SCN5A* (+) patients with symptoms at diagnosis had a higher risk of arrhythmic events during follow-up in comparison to *SCN5A* (–) patients with symptoms at diagnosis. However, an opposite result was demonstrated by a recent study. It reported that 28 variants in *SCN5A* and other 9 genes in Human Gene Mutation Database were identified to be related to BrS, whereas, neither type 1 BrS ECG pattern nor abnormal J-point elevation in V1 and V2 was observed among genes mutations carriers. Besides, no difference was noted in susceptibility of syncope, ventricular cardiac events, or entirety mortality (Ghouse et al., 2017). On the contrary, Hosseini et al found that *SCN5A* was the only gene which is clinically associated with BrS among 21 included gene (Hosseini et al., 2018), thus, further studies needed to be done.

On the other hand, there was no significant difference between *SCN5A* (+) patients and *SCN5A* (–) patients who were asymptomatic at diagnosis. This may be due to the presence of a BrS-like ECG pattern in some cases including serious coronary events, imbalanced electrolyte, pharmacologic factors, pulmonary embolism, right bundle branch block, arrhythmogenic right ventricular cardiomyopathy, abnormalities in autonomic nervous system, and left ventricular hypertrophy (Shi et al., 2018). According to the specific situation, a certain number of patients might be wrongly diagnosed.

It is worth mentioning that in the meta-analysis, I^2^ was zero, which indicates that there was no analytical bias caused by a single dataset. Negative results were obtained according to the original data for each study, but a positive result was obtained when the sample size was expanded. This is the first time that *SCN5A* (+) is reported to increase the risk of future heart events among BrS patients in patients with symptoms at diagnosis.

### EPS Groups

According to the 2017 guideline for ventricular cardiac events and SCD, further risk stratification in asymptomatic and spontaneous type 1 patients with EPS following programmed ventricular stimulation using single or double extrastimuli could be considered (Al-Khatib et al., 2018). On the other hand, the current guideline demonstrated that EPS inducibility appeared in a large number of BrS patients who suffered from previous sudden death or syncope (Priori et al., 2013). In addition, PES was of value particularly in patients with previous syncope: in this group, PES aided in the prevention of more than half of unnecessary ICD implants, when there was a follow-up at least within a mean of 30 months (Giustetto et al., 2009).

In the present study, we focused on the relationship between EPS and *SCN5A* gene mutation status. While few studies have investigated this relationship, negative conclusions were noted in Yamagata et al. (2017) and Andorin et al. (2016). Therefore, we performed a related analysis including 554 BrS patients who underwent EPS from three studies and obtained a positive result. The present analysis revealed that *SCN5A* (+) BrS patients with EPS negative probably have a higher prevalence of subsequent arrhythmia events, however, the risk of *SCN5A* (+) was not higher than that of *SCN5A* (–) in patients with EPS (+) BrS. This suggests that *SCN5A* may contribute to the occurrence of future events. According to previous studies, *SCN5A* (+) BrS patients had a longer HV interval during EPS compared to *SCN5A* (–) BrS patients or purely EPS positive BrS patients. This implies that the underlying electrophysiologic mechanisms of conduction block and ventricular arrhythmia are strongly correlated (Giustetto et al., 2009; Yamagata et al., 2017).

## Study Limitation and Conclusions

In this study, the number of patients who underwent genetic testing was still limited, probably due to the high cost of the test. Secondly, the inadequacy of the original data prevented further analysis. In addition, *SCN5A* mutations can be variable with presumably differing effects on sodium channel function. Nevertheless, this study shows that positive mutation status is an important determinant of outcomes in particular subgroups described above. Finally, the relationship between BrS patients with symptoms and the higher frequency of future events needs to be further enhanced and additional experiments are required. *SCN5A* (+) as a risk marker of BrS should not be underestimated. *SCN5A* (+) patients with symptoms at diagnosis may have a prognosis significance for BrS. Furthermore, *SCN5A* (+) BrS patients with EPS (−) displayed a higher prevalence of future cardiac events.

## Author Contributions

All authors listed have made a substantial, direct and intellectual contribution to the work, and approved it for publication.

### Conflict of Interest Statement

HB-M was employed by company Global Genetics Corp, Ventura, California, USA. The remaining authors declare that the research was conducted in the absence of any commercial or financial relationships that could be construed as a potential conflict of interest.
