# Supplementary material for: Meta-Analysis of Risk Stratification of SCN5A With Brugada Syndrome: Is SCN5A Always a Marker of Low Risk?
Source: Front Physiol. 2019 Feb 19;10:103. doi: 10.3389/fphys.2019.00103 (PMC6389868; doi:10.3389/fphys.2019.00103)
Supplement: Supplementary file 1 [file Image_1.pdf]

Supplementary

Figure 1. Odds ratio for the occurrence of arrhythmic events during follow-up among BrS patients who

Study or Subgroup	SCN5A Mutation positive Events	Total	SCN5A Mutation negative Events	Total	Weight	Odds Ratio M–H, Fixed, 95% CI	Year
Gasparini et al.2002	0	8	0	13		Not estimable	2002
Juang et al.2003	1	1	2	3	1.2%	1.80 [0.04, 79.42]	2003
Mok et al.2004	2	5	18	31	8.8%	0.48 [0.07, 3.30]	2004
Liang et al.2006	1	1	3	3		Not estimable	2006
Yuan 2008	0	1	4	6	4.4%	0.19 [0.01, 6.48]	2008
Priori et al.2012	3	24	6	99	6.0%	2.21 [0.51, 9.58]	2012
Sacher et al.2013	6	41	16	119	20.6%	1.10 [0.40, 3.04]	2013
Tokioka et al.2014	4	17	13	106	8.1%	2.20 [0.62, 7.78]	2014
Andorin et al.2016	9	58	0	17	1.9%	6.72 [0.37, 121.54]	2016
Calò et al.2016	2	32	10	75	16.5%	0.43 [0.09, 2.10]	2016
Yamagata et al.2017	13	60	49	355	32.6%	1.73 [0.87, 3.42]	2017
**Total (95% CI)**		**248**		**827**	**100.0%**	**1.37 [0.89, 2.11]**	
Total events	41		121				

Heterogeneity: $Chi^2 = 7.14$, df = 8 (P = 0.52); $I^2 = 0\%$
Test for overall effect: Z = 1.44 (P = 0.15)

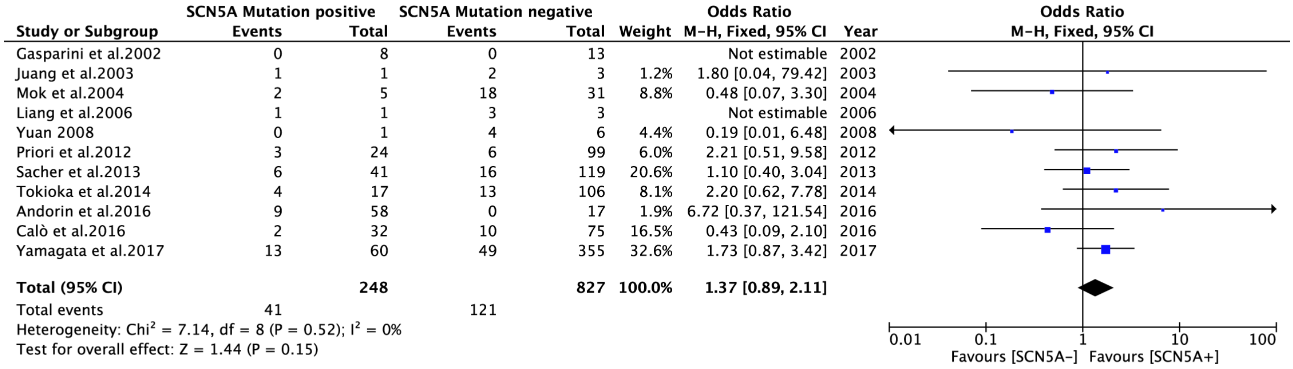

underwent *SCN5A* gene mutation test.
